# Cardiovascular safety of non-insulin pharmacotherapy for type 2 diabetes

**DOI:** 10.1186/s12933-017-0499-5

**Published:** 2017-02-02

**Authors:** James Xu, Rohan Rajaratnam

**Affiliations:** 1 Cardiology Department, Level 1 CSB, Liverpool Hospital, Elizabeth Street, Liverpool, NSW 2170 Australia; 2 Cardiology Department, Campbelltown Hospital, Sydney, NSW Australia; 3 South Western Sydney Clinical School, The University of New South Wales, Sydney, NSW Australia; 4 Macarthur Clinical School, Western Sydney University, Parramatta, NSW Australia

**Keywords:** Type 2 diabetes, Anti-diabetic drugs, Cardiovascular outcome trials

## Abstract

Patients with type 2 diabetes mellitus have a twofold increased risk of cardiovascular mortality compared with non-diabetic individuals. There is a growing awareness that glycemic efficacy of anti-diabetic drugs does not necessarily translate to cardiovascular safety. Over the past few years, there has been a number of trials evaluating the cardiovascular effects of anti-diabetic drugs. In this review, we seek to examine the cardiovascular safety of these agents in major published trials. Metformin has with-stood the test of time and remains the initial drug of choice. The sulfonylureas, despite being the oldest oral anti-diabetic drug, has been linked to adverse cardiovascular events and are gradually being out-classed by the various other second-line agents. The glitazones are contraindicated in heart failure. The incretin-based drugs have been at the fore-front of this era of cardiovascular safety trials and their performances have been reassuring, whereas the meglitinides and the alpha-glucosidase inhibitors still lack cardiovascular outcomes data. The sodium glucose cotransporter-2 inhibitors are an exciting new addition that has demonstrated a potential for cardiovascular benefit. Many of the currently available oral anti-diabetic agents have clinically relevant cardiovascular effects. The optimal approach to the reduction of cardiovascular risk in diabetic patients should focus on aggressive management of the standard cardiovascular risk factors rather than purely on intensive glycemic control.

## Background

Cardiovascular disease remains the leading cause of morbidity and mortality in type 2 diabetes mellitus (T2DM). Despite a decline in the rate of cardiovascular mortality over time, men and women with diabetes mellitus remain at twofold increased risk [1]. However, in patients with long-standing T2DM, glucose lowering does not necessarily reduce adverse cardiovascular outcomes, as demonstrated by several large-scale randomized studies [2–4]. In fact, the action to control cardiovascular risk in diabetes (ACCORD) trial was terminated early due to a 22% excess in cardiovascular mortality in the intensive glycemic control group [2]. Although the United Kingdom Prospective Diabetes Study (UKPDS) showed that glucose lowering reduced macrovascular complications in patients with newly diagnosed T2DM, this was only observed after median follow-up period of more than 15 years [5].

In 2007, the highly publicized controversy surrounding the cardiovascular safety of rosiglitazone reinforced a growing awareness that glycemic efficacy is no longer the sole determinant of anti-diabetic drug pre-approval trials [6]. This led to a major change in United States Food and Drug Administration (FDA) policy in 2008, resulting in trials involving newer anti-diabetic drugs to emphasize cardiovascular safety [6].

The increasing prevalence of T2DM worldwide will likely be paralleled by a rise in prevalence of cardiovascular disease. With the numerous anti-diabetic drugs available, there is a pressing need to clearly define their potential cardiovascular effects. Here, we review the contemporary literature examining the cardiovascular benefits and risks of currently available non-insulin anti-diabetic drugs.

## Biguanides

### Metformin

Metformin, along with the sulfonylureas, is one of two oral anti-diabetic drug groups listed on the World Health Organization Model Lists of Essential Medicines [7]. It inhibits hepatic gluconeogenesis and increases insulin-mediated glucose uptake in peripheral tissues [8]. Metformin reduces mean glycated hemoglobin (HbA1c) by approximately 1.5% compared to placebo [9], and promotes weight loss of up to 2.5 kg, sustainable over 10 years [10]. Long term therapy with metformin has also been shown to beneficially alter the lipid profile, reducing serum triglyceride and plasma total cholesterol levels and slightly increasing serum high-density-lipoprotein cholesterol levels [11]. Metformin may also have vascular protective effects through increased angiogenesis by improving the angiogenic potential of CD34^+^ cells [12]. Furthermore, the potential beneficial actions of metformin on endothelial function have also been reported. For instance, metformin improved circulating endothelial progenitor cells in diabetic patients [13], as well as bone marrow endothelial progenitor cell functions in streptozotocin-induced diabetic mice [14].

#### Cardiovascular safety

The UKPDS showed that metformin monotherapy in obese newly diagnosed T2DM patients was associated with a 32% reduction in the aggregate diabetes-related endpoint, including sudden death and myocardial infarction (MI) (p = 0.011), and 36% reduction in all-cause mortality (p = 0.011) [15]. This benefit was sustainable, with persistent risk reductions observed in the metformin group for any diabetes-related endpoint (21%, p = 0.01), MI (33%, p = 0.005) and all-cause mortality (27%, p = 0.002), during 10 years of post-trial follow-up of the UKPDS survivor cohort [5]. A meta-analysis of 35 randomized trials showed that metformin reduced adverse cardiovascular events (MI, stroke, peripheral artery disease and cardiovascular death) versus placebo/no therapy (MH-OR 0.79, 95% CI 0.64–0.98, p = 0.031), but not against active-comparators (MH-OR 1.03, 95% CI 0.72–1.77, p = 0.89) [16]. A non-significant trend towards reduced all-cause mortality was also noted with metformin monotherapy (MH-OR 0.801, 95% CI 0.625–1.024, p = 0.076) [16]. This benefit may be especially pronounced in obese patients, with a systematic review of 29 trials reporting a reduction in all-cause mortality with metformin in this population when compared with chlorpropamide, glyburide and insulin [17].

Metformin is not contraindicated in heart failure, although there has been concerns regarding its safety in this setting due to reports of increased incidence of lactic acidosis [18]. A systematic review of 9 cohort studies found no increased risk for morbidity and mortality with metformin use across various subgroups of patients with heart failure, including those with reduced left ventricular ejection fraction or concomitant chronic kidney disease [19].

Metformin use is restricted in the setting of iodinated contrast-enhanced procedures again due to a presumed association with lactic acidosis. However, an analysis of the Prevention of Restenosis with Tranilast and its Outcomes (PRESTO) trial showed that diabetic patients undergoing coronary angioplasty who were treated with metformin had fewer rates of death (OR 0.39, 95% CI 0.19–0.77, p = 0.007) and MI (OR 0.31, 95% CI 0.15–0.66, p = 0.002) compared to other anti-diabetic therapies [20]. Due to paucity of randomized evidence, guidelines vary substantially on their recommendations regarding the timing of discontinuation of metformin prior to contrast exposure [21, 22].

#### Summary

Metformin has an excellent cardiovascular safety profile, even with long-term use. Given its low cost, efficacy and proven safety record, guidelines recommend metformin as the first-line drug therapy of choice for T2DM [23, 24]. Lactic acidosis is a rare but potentially fatal adverse effect. However, with an average estimated incidence of less than 0.03 per 1000 patient-years, this risk is minimal, and predominantly occurs in the setting of severe renal insufficiency [11].

## Insulin secretagogues

### Sulfonylureas

The sulfonylureas are the oldest and most widely prescribed oral anti-diabetic agents [25]. They bind to receptors on adenosine triphosphate-sensitive potassium channels (K_ATP_) in the pancreatic β-cells, causing depolarization of the cell membrane and subsequent calcium-mediated exocytosis of insulin-containing secretory granules [26]. First-generation sulfonylureas such as tolbutamide and chlorpropamide are no longer used due to high incidence of adverse reactions [25]. Second-generation agents include glipizide, glibenclamide (also known as glyburide in United States) and gliclazide. Sulfonylurea monotherapy reduces HbA1c by 1–2% [27], but weight gain is almost inevitable [25]. In the UKPDS, patients randomized to sulfonylureas gained a mean of 5.3 kg over 6 years [28]. Glimepiride, a third-generation agent, has at least therapeutic equivalence to the second-generation agents, but is less associated with both weight gain and hypoglycemia [29], and may have lesser undesirable effects on myocardial preconditioning due to its selectivity to pancreatic K_ATP_ [30].

#### Cardiovascular safety

The University Group Diabetes Study (UGDS) first reported that T2DM patients randomized to tolbutamide had increased cardiovascular mortality compared with placebo or insulin [31]. Several subsequent observational studies supported an association between sulfonylurea use and increased cardiovascular mortality [32–34]. Recent meta-analyses reported inconsistent findings. A review of 115 randomized trials found a 22% increased risk of all-cause mortality with sulfonylurea therapy compared with placebo or other anti-diabetic drugs (pooled-OR 1.22, 95% CI 1.01–1.49), although the overall incidence of major adverse cardiovascular events (MACE) appeared to be unaffected [35]. In contrast, a Cochrane review of 72 randomized trials reported no significant association between sulfonylureas and mortality compared with metformin monotherapy (pooled RR 1.47, 95% CI 0.54–4.01) [27], despite the possible protective effects of metformin. Interference with protection from ischemic preconditioning due to blockade of mitochondrial K_ATP_ may contribute to the observed association between sulfonylureas and cardiovascular mortality, although glibenclamide pre-treatment did not abrogate the protective effect of glucagon-like peptide-1 (GLP-1) in human models of non-lethal myocardial ischemia [36].

The impact of sulfonylureas on cardiovascular outcomes may not be a class effect. There is evidence that whilst first-generation sulfonylureas were associated with increased cardiovascular mortality compared to placebo (RR 2.63, 95% CI 1.32–5.22, p = 0.006), no difference was found for second-generation agents [27]. A review of 18 randomized trials showed that gliclazide was associated with a lower risk of all-cause (RR 0·65, 95% CI 0·53–0·79) and cardiovascular-related mortality (RR 0·60, 95% CI 0·45–0·84) compared with glibenclamide [37], suggesting a possible benefit specific for gliclazide.

#### Summary

The newer sulfonylureas (gliclazide and glimepiride) may have a more favorable cardiovascular effect profile than older agents and should be the preferred agents in this class. Overall, due to their low cost and short-term glycemic efficacy, the sulfonylureas are still strongly endorsed as second-line agents by international guidelines [23, 24].

### Meglitinides

The meglitinides have similar action to sulfonylureas but are pharmacologically distinct and bind to different receptors on pancreatic K_ATP_ channels [38]. Currently available meglitinides include repaglinide and nateglinide. They exert similar but milder clinical effects compared to sulfonylureas [39]. A Cochrane review of 15 randomized trials showed that repaglinide was more efficacious in HbA1c reduction than nateglinide (0.1–2.1% for repaglinide, 0.2–0.6% for nateglinide). These agents also cause weigh gains of up to 3 kg in 3 months [40].

#### Cardiovascular safety

There are currently no long-term studies of meglitinides to assess cardiovascular outcomes or mortality in T2DM, although the Nateglinide and Valsartan in Impaired Glucose Tolerance Outcomes Research (NAVIGATOR) study may provide some insight. This was a large 2 × 2 factorial randomized trial of nateglinide versus placebo, and valsartan versus placebo in 9306 patients with impaired glucose tolerance and cardiovascular disease or risk factors. After a median follow up of 5 years, there was no difference between nateglinide and placebo with respect to the core composite cardiovascular outcome of cardiovascular death, MI, stroke or heart failure hospitalization (7.9 vs 8.3%, HR 0.94, 95% CI 0.82–1.09, p = 0.43), or the extended composite cardiovascular outcome, including arterial revascularization and hospitalization for unstable angina (14.2 vs 15.2%, HR 0.93; 95% CI 0.83–1.03, p = 0.16) [41]. A Danish nation-wide registry-based observational analysis showed that mortality and cardiovascular risk associated with the use of repaglinide, and gliclazide, was similar to metformin [42].

#### Summary

Meglitinides are associated with less hypoglycemia and weight gain compared with sulfonylureas [43]. However, due to the paucity of cardiovascular outcomes data, these agents should be reserved in favor of other anti-diabetic drugs.

## Thiazolidinediones

The thiazolidinediones (TZDs), also known as glitazones, are a class of potent insulin-sensitizers which act by regulating gene expression through selective ligand-binding of the nuclear transcription factor peroxisome-proliferator–activated receptor ɣ (PPARɣ) [44]. Rosiglitazone and pioglitazone are the currently approved agents in this class. The TZDs lowers HbA1c by 1–1.5% on average in placebo controlled studies, with low risk of hypoglycemia [44]. An increase in body weight of 2–3 kg for every 1% decrease in HbA1c is expected [44]. However, this weight gain is partly due to a redistribution of visceral fat to subcutaneous adipose tissue, and therefore may not be entirely undesirable [44]. Pioglitazone compared with rosiglitazone is associated with significant improvements in triglyceride and cholesterol profiles [45].

### Cardiovascular safety

In a meta-analysis of 42 randomized trials, many of which were unpublished clinical trial registry data, rosiglitazone was associated with a significant increase in the risk of MI (OR 1.43, 95% CI 1.03–1.98, p = 0.03) and a non-significant trend for increased cardiovascular mortality (OR 1.64, 95% CI 0.98–2.74, p = 0.06) [46]. This highly-publicized study resulted in a boxed warning of myocardial ischemia for rosiglitazone in 2007 [6]. In response, an interim analysis of the Rosiglitazone Evaluated for Cardiac Outcomes and Regulation of Glycemia in Diabetes (RECORD) trial was published. This trial randomized 4447 T2DM patients to rosiglitazone plus either metformin or sulfonylurea or an active control (metformin plus sulfonylurea). No elevated risk for MI or death in the rosiglitazone group was noted at 3.75 years follow-up [47]. The final analysis showed that after a mean follow up of 5.5 years, rosiglitazone was non-inferior to a combination of metformin and sulfonylurea with regards to the primary endpoint of cardiovascular hospitalization or cardiovascular death (HR 0·99, 95% CI 0·85–1·16), but its effect on MI was inconclusive due to small number of events (HR 1·14, 95% CI 0·80–1·63) [48].

Pioglitazone was compared with placebo in the Prospective Pioglitazone Clinical Trial in Macrovascular Events (PROACTIVE) study, which randomized 5238 T2DM patients at high risk for macrovascular complications. The trial was terminated prematurely after an average follow up of 34.5 months due to significant reduction in the main secondary composite endpoint of all-cause mortality, non-fatal MI, and stroke in the pioglitazone group (HR 0·84, 95% CI 0·72–0·98, p = 0·027). Pioglitazone also non-significantly reduced the composite primary endpoint of all-cause mortality and various cardiovascular outcomes, including MI, stroke, and vascular interventions (HR 0·90, 95% CI 0·80–1·02, p = 0·095) [49]. A meta-analysis of 19 randomized trials also showed a lower risk of death, MI or stroke with pioglitazone therapy compared to placebo or active comparator (4.4% v 5.7%, HR 0.82, 95% CI 0.72–0.94, p = 0.005) [50].

TZD use has consistently been associated with increased risk of heart failure, as shown in a meta-analysis of 29 placebo-controlled trials (5.3 vs 3.7%, OR 1.59, 95% CI 1.34–1.89, p < 0.00001) [51]. In RECORD, rosiglitazone was associated with increased risk of fatal and non-fatal heart failure (HR 2.10, 95% CI 1.35–3.27) [48]. Similar findings were noted for pioglitazone in PROACTIVE (HR 1.43, 95% CI 1.13–1.81) [49]. The Diabetes Reduction Assessment with Ramipril and Rosiglitazone Medication (DREAM) trial also showed that rosiglitazone therapy led to an increase in non-fatal heart failure in patients with impaired glucose tolerance (HR 7·03, 95% CI 1.6–30.9, p = 0.01) [52].

### Summary

Current data suggests increased caution with rosiglitazone use. In contrast, pioglitazone may confer cardiovascular benefits. There is no doubt however that both agents should be avoided in patients with or at risk for heart failure, and this is reflected in the latest European heart failure guidelines as a class IIIA recommendation [53]. Moreover, adverse side effects and relatively high drug costs limit the widespread use of TZDs as monotherapy, and they are more appropriately considered complementary to other oral anti-diabetic drugs. To receive maximum benefits, patients should have repeated body weight measurements and periodic liver function tests [54].

## Incretin-based drugs

### Dipeptidyl peptidase-4 inhibitors

The incretins, namely glucose-dependent insulinotropic peptide (GIP) and GLP-1, are insulinotropic gut hormones that modulate the insulin secretory response to food intake in a glucose-dependent manner. They are rapidly degraded by circulating enzymes called dipeptidyl peptidase-4 (DPP-4) [55]. There are currently 4 FDA-approved DPP-4 inhibitors: sitagliptin, saxagliptin, linagliptin and alogliptin. Vildagliptin is not available in United States. A meta-analysis reported 0.74% HbA1c reduction when treatment with sitagliptin and vildagliptin was compared to placebo [56], and this glycemic efficacy appears to be equivalent across the class [57]. The DPP-4 inhibitors, otherwise known as the gliptins, are generally considered to have a neutral effect on weight [58]. It has been suggested that the modulation of endothelial progenitor cells, inflammatory pathways and ischemic response are the major cardiovascular targets of gliptins [59].

#### Cardiovascular safety

The Saxagliptin Assessment of Vascular Outcomes Recorded in Patients with Diabetes Mellitus—Thrombolysis in Myocardial Infarction (SAVOR-TIMI 53) trial randomized 16,492 patients with T2DM and either a history of cardiovascular disease or multiple vascular risk factors to receive saxagliptin or placebo on top of conventional therapy. After a median follow-up of 2.1 years, there was no difference in the primary composite endpoint of cardiovascular death, non-fatal MI, or non-fatal ischemic stroke between groups (7.3 vs 7.2%, HR 1.00, 95% CI 0.89–1.12, p = 0.99 for superiority, p < 0.001 for non-inferiority). However, in the pre-specified secondary end-points analysis, heart failure hospitalization was more common in the saxagliptin group (3·5 vs 2·8%, HR 1·27, 95% CI 1·07–1·51, p = 0·007) [60], although this increased risk subsided by 10–11 months after randomization [61].

In the Examination of Cardiovascular Outcomes with Alogliptin versus Standard of Care (EXAMINE) trial, which randomized 5380 patients with T2DM and a recent acute coronary syndrome in the last 15–90 days, alogliptin was non-inferior to placebo with regards to the combined primary outcome of cardiovascular death, non-fatal MI or non-fatal stroke after a median follow up of 18 months (11.3 vs 11.8%, HR 0.96, upper boundary of one sided repeated CI 1.16; p < 0.001 for non-inferiority) [62]. A post hoc analysis showed that alogliptin had no effect on heart failure outcomes, including a composite of cardiovascular death and heart failure hospitalization (HR 1.00, 95% CI 0.82–1.21) [63].

Similarly, in the trial to evaluate cardiovascular outcomes after treatment with sitagliptin (TECOS) study, which randomized 14,671 patients with T2DM, sitagliptin was non-inferior to placebo for the primary composite outcome of cardiovascular death, non-fatal MI, non-fatal stroke, or hospitalization for unstable angina (HR 0.98, 95% CI 0.88–1.09, p < 0.001), with no difference in hospitalization rates for heart failure (HR 1.00, 95% CI 0.83–1.20, p = 0.98) [64]. Sitgaliptin has also been shown to reduce blood pressure and improve albuminuria in a prospective study of Japanese patients with T2DM [65]. In addition, long-term use of sitagliptin up to 2 years did not adversely alter endothelial function in patients with T2DM [66].

The cardiovascular safety profile of linagliptin was investigated in a large patient-level pooled safety analysis of 19 clinical trials, which concluded that linagliptin was not associated with increased cardiovascular risk versus pooled active comparators or placebo in patients with T2DM, irrespective of background therapy [67].

Mostly driven by the results of the large SAVOR trial, a meta-analysis concluded that there was a slightly increased risk of hospitalization for heart failure in gliptin users compared with placebo (3.4 vs 3.0%, OR 1.13, 95% CI 1.00–1.26) [68]. However, the Canadian Network for Observational Drug Effect Studies (CNODES) retrospectively analyzed administrative electronic health records of nearly 1.5 million patients and concluded that DPP-4 inhibitors did not increase the rate of heart failure hospitalization compared with oral anti-diabetic drug combinations among patients with (13.8 vs 11.6%, HR 0.87, 95% CI, 0.63–1.21) or without a history of heart failure (9.6 vs 8.9%, HR 0.84, 95% CI 0.69–1.02) [69]. Another large scale cohort study analyzed an FDA database covering 358 million person-years of observation, also found no increased risk of heart failure hospitalizations in users of saxagliptin or sitagliptin compared with other selected anti-diabetic agents [70]. Furthermore, patients treated with gliptins in a comprehensive national cohort of diabetic patients in Taiwan had lower risks of cardiovascular disease, including heart failure, compared to non-gliptin users, except for metformin [71].

#### Summary

The DPP-4 inhibitors are safe in terms of hard cardiovascular endpoints, but their effect on the risk of heart failure remains uncertain. The clinical significance of the finding of an early increased hospitalization for heart failure with saxagliptin in the SAVOR study is unclear, although it is unlikely to be a class effect. The FDA safety review recommends considering discontinuation of specifically saxagliptin or alogliptin in patients who develops heart failure [72].

### Glucagon-like peptide-1 agonists

The GLP-1 agonists are parenterally administered drugs that directly activate the GLP-1 receptor and are highly resistant to degradation by DPP-4. Currently available agents include: exenatide, liraglutide, albiglutide, lixisenatide, and dulaglutide. All are FDA-approved with the exception of lixisenatide, which is approved in Europe. GLP-1 agonists, when compared to placebo, reduced HbA1c by about 1% and resulted in 1.5–2.5 kg weight loss over 30 weeks [73]. In addition, treatment with GLP-1 agonists have been shown to further favorably alter the metabolic profile through modest reductions in low-density-lipoprotein cholesterol, total cholesterol and triglycerides [74], as well as reductions in systolic blood pressure (weighted mean difference −2.22 mmHg; 95% CI −2.97 to −1.47), although this may be accompanied by a compensatory increase in heart rate [75].

#### Cardiovascular safety

The cardioprotective effects of GLP-1 agonists have been well documented in pre-clinical studies. Indeed, experimental models of ischemia and reperfusion have shown improved post-ischemic myocardial contractile dysfunction and reduced infarct size with constant infusions of GLP-1 [76]. In the clinical setting, similar positive effects have been observed to various degrees in pilot studies, and the mechanism was thought to be related to reduced apoptosis and nuclear oxidative stress and improvement in myocardial glucose metabolism [77].

The Evaluation of Lixisenatide in Acute Coronary Syndrome (ELIXA) trial randomized 6068 patients with T2DM and an acute coronary event within the last 180 days to receive lixisenatide or placebo on top of standard of care. After a median follow-up of 25 months, there was no difference in the primary composite endpoint of cardiovascular death, non-fatal MI, non-fatal stroke, or hospitalization for unstable angina between groups (13.4 vs 13.2%, HR 1.02, 95% CI 0.89–1.17, p = 0.81 for superiority, p < 0.001 for non-inferiority), and no difference in heart failure hospitalizations (4.0 vs 4.2%, HR 0.96, 95% CI 0.75–1.23) [78].

In the Liraglutide Effect And Action In Diabetes: Evaluation Of Cardiovascular Outcome Results (LEADER) trial, which randomized 9340 T2DM patients with high cardiovascular risk, liraglutide reduced the primary composite endpoint of cardiovascular death, non-fatal MI or non-fatal stroke compared to placebo after a median follow-up of 3.8 years (13 vs 14.9%, HR 0.8, 95% CI 0.78–0.97, p < 0.001 for non-inferiority; p = 0.01 for superiority). All-cause mortality was also reduced with liraglutide (8.2 vs 9.6%, HR 0.85, 95% CI 0.74–0.97, p = 0.02). The placebo group was more likely to receive added insulin or sulfonylureas, and consequently had a higher incidence of severe hypoglycemia (3.3 vs 2.4%, p = 0.02) [79]. In another study of 41 newly diagnosed T2DM patients with stable coronary artery disease, although 12 weeks of liraglutide treatment, when compared to placebo, did not improve left ventricular ejection fraction, there was no increased risk of hospitalization for heart failure [80].

Although not currently FDA-approved, semaglutide, a once-weekly GLP-1 agonist, was non-inferior to placebo after 26 months follow-up among 3297 patients with T2DM in the Trial to Evaluate Cardiovascular and Other Long-term Outcomes with Semaglutide in Subjects with Type 2 Diabetes (SUSTAIN-6) with regards to the primary composite endpoint of cardiovascular death, non-fatal MI and non-fatal stroke (HR 0.74, 95% CI 0.58–0.95, p < 0.001 for non- inferiority) [81]. Notably, majority of the trial patients had established cardiovascular disease, stage 3 or higher chronic kidney disease, or both at baseline.

Despite the lack of definitive randomized data, dulaglutide, another once-weekly long-acting GLP-1 agonist, was not associated with an increased risk of MACE versus comparator therapy in a meta-analysis of phase 2 and phase 3 trials [82].

The retrospective CNODES study also showed that GLP-1 agonists did not increase heart failure hospitalization among patients with (0.5 vs 0.7%, HR 0.75, 95% CI, 0.22–2.51) or without a history of heart failure (1.0 vs 0.9%, HR 0.95, 95% CI 0.83–1.10) [69]. In fact, GLP-1 infusion has been associated with improvements in left ventricular function in heart failure in both pre-clinical and clinical settings [77].

#### Summary

The LEADER trial is encouraging, but the outcomes may be biased in favor of liraglutide due to the detrimental effects of added anti-diabetic drugs in the placebo group. More long-term studies of GLP-1 agonists to assess cardiovascular outcomes as well as the durability of weight loss are needed.

## Sodium glucose cotransporter-2 inhibitors

The sodium glucose cotransporter-2 (SGLT-2) is expressed in the proximal tubule and mediates reabsorption of up to 98% of urinary glucose [83]. Inhibition of SGLT-2 lowers blood glucose by promoting renal glucose excretion, which is independent of β-cell function and thus carries a low risk for hypoglycemia [84]. Currently, 3 agents are approved in the United States and Europe: dapagliflozin, canagliflozin, and empagliflozin. SGLT-2 inhibitors reduce HbA1c by 0.5–0.7% compared with placebo [85, 86], and promotes weight loss of 2–3 kg over 12 weeks [83, 87]. It has a modest beneficial effect on the lipid profile through an increase in high-density-lipoprotein cholesterol and decrease in triglycerides [87]. In part due to the osmotic diuresis, SGLT-2 inhibitors also significantly reduce both systolic (weighted mean difference −4.0 mmHg; 95% CI −4.4 to −3.5) and diastolic blood pressure (weighted mean difference −1.6 mmHg; 95% CI −1.9 to −1.3), without an increase in heart rate [88]. Furthermore, empagliflozin has been shown in preclinical animal studies to prevent the development of endothelial dysfunction and reduce oxidative stress [89].

### Cardiovascular safety

In the only cardiovascular outcomes trial of SGLT-2 inhibitors reported to date, the randomized empagliflozin cardiovascular outcome event trial in type 2 diabetes mellitus patients (EMPA-REG OUTCOME) trial of 7020 patients with T2DM and established cardiovascular disease showed that after a median follow up of 3.1 years, empagliflozin was associated with a reduction in the primary composite endpoint of cardiovascular mortality, non-fatal MI, or non-fatal stroke compared with placebo (10.5 vs 12.1%, HR 0.86, 95% CI 0.74–0.99, p = 0.04 for superiority, NNT 62). A significant reduction in all-cause mortality (5.7 vs 8.3%, p < 0.001, NNT 38) and cardiovascular mortality (3.7 vs 5.9%, p < 0.001, NNT 45) was also observed in the empagliflozin group. Further analysis showed a 34% relative risk reduction of a composite of heart failure hospitalization or cardiovascular death with empagliflozin (5.7 vs 8.5%, HR 0.66, 95% CI 0.55–0.79, p < 0.001, NNT 35) [90]. Driven mainly by these results, a meta-analysis of data from regulatory submissions and published trials suggested net protection of SGLT-2 inhibitors against cardiovascular outcomes and death [91]. Furthermore, empagliflozin has an excellent long term safety and tolerability profile. A study of empagliflozin monotherapy for ≥76 weeks in T2DM patients showed a lower rate of drug discontinuation due to adverse events compared with placebo [92].

A meta-analysis of phase 2 and phase 3 trials showed that dapagliflozin was not associated with an increased risk of MACE compared with placebo or comparator therapy in T2DM patients, and this observation was consistent among patients with varying degrees of cardiovascular risk [93].

### Summary

The results from the EMPA-REG trial are very exciting. Although long-term clinical experience is still required, the SGLT-2 inhibitors is currently favored as the second-line agent of choice in T2DM patients with a history of cardiovascular disease [94]. Additional data from planned and continuing studies of SGLT-2 inhibitors are eagerly awaited.

## Alpha glucosidase inhibitors

The alpha glucosidase inhibitors (AGIs) lower blood glucose through competitive blockade of intestinal alpha-glucosidases, which convert complex carbohydrates into monosaccharides. This results in a modified intestinal absorption of carbohydrates and consequently a slower rise in post-prandial blood glucose [95]. Available agents include acarbose, miglitol and voglibose. A Cochrane meta-analysis reported a 0.8% HbA1c reduction and no clinically relevant effects on lipids or body weight when acarbose, the most widely prescribed AGI, was compared to placebo [96]. In addition, acarbose may also have beneficial effects on endothelial function by obtunding post-prandial glucotoxicity [95]. Miglitol, whilst having similar glycemic efficacy, may have a greater effect on reducing 1 h post-prandial glucose levels than other AGIs [97]. A study of 47 Japanese patients with T2DM showed that switching from other AGIs to miglitol for 3 months significantly improved glucose fluctuations and reduced serum concentrations of inflammatory cytokines [98]. Miglitol has also been shown to reduce waist circumference, and in particular visceral fat, in patients with metabolic syndrome [99].

### Cardiovascular safety

There are no long-term studies examining the effect of AGIs on cardiovascular disease or mortality in T2DM. The Study to Prevent Non-insulin-dependent Diabetes Mellitus (STOP-NIDDM) trial randomized 1429 patients with impaired glucose tolerance and showed that after a mean follow-up of 3.3 years, acarbose treatment was associated with a significant risk reduction in the development of diabetes (HR 0.75, 95% CI 0.63–0.90, p = 0.0015) [100] and hypertension (HR 0.66, 95% CI 0.49–0.89, p = 0.006) [101], compared to placebo. Although not initially powered to draw conclusions on cardiovascular outcomes, acarbose treatment was also associated with a reduction in the development of the composite outcome of cardiovascular events, which includes cardiovascular death, MI, stroke, heart failure, peripheral vascular disease and revascularization (HR 0.51, 95% CI 0.28–0.95, p = 0.03) [101].

### Summary

Long-term cardiovascular outcomes data is lacking for the AGIs in T2DM and therefore these cannot be recommended over the other available agents. However, treatment is associated with minimal adverse effects and low risk of hypoglycemia.

## Conclusions

Due to a high prevalence of cardiovascular morbidity and mortality in T2DM, the optimal approach to the reduction of cardiovascular risk should focus on aggressive management of the standard cardiovascular risk factors rather than purely on intensive glycemic control. As can be seen from the various trials reviewed here, favorable glycemic efficacy does not necessarily translate to favorable cardiovascular outcomes (Table 1). Clinicians must therefore make careful informed decisions based on the cardiovascular effects of the various anti-diabetic drugs when prescribing (Table 2). Based on current evidence, metformin should remain the first-line drug of choice in T2DM, being the most extensively studied and demonstrating excellent cardiovascular safety even with long term use. Although evidence for the cardiovascular safety of sulfonylureas are inconsistent, the first-generation agents are probably associated with net harm and should be avoided. Newer generation sulfonylureas have a comparatively more favorable cardiovascular profile, but weight gain remains a concern. The meglitinides and AGIs lack cardiovascular safety data in T2DM and should therefore be reserved in favor of other second-line agents. Among the TZDs, rosiglitazone may be associated with an increased risk of MI, while pioglitazone may have beneficial cardiovascular effects. Both are however contraindicated in heart failure. The incretin-based drugs have been at the forefront of this era of cardiovascular safety trials and have been extensively studied. Current evidence suggests that the gliptins have neutral overall cardiovascular effect, but may increase risk of heart failure, particularly saxagliptin. Among the GLP-1 agonists, liraglutide may have beneficial effects on cardiovascular outcomes, but this requires further validation. Similarly, the SGLT-2 inhibitors have shown promising results with empagliflozin and may potentially confer cardiovascular benefits, although additional data is needed to substantiate this. With results of several large ongoing randomized trials expected in the coming years (Table 3), the body of evidence will continue to expand and help guide clinicians in making the best decision in reducing the cardiovascular risk of their diabetic patients.

**Table 1:** Comparison of the various pharmacotherapy for type 2 diabetes mellitus

	Metformin	Sulfonylureas		Meglitinides	TZDs		DPP-4 inhibitors	GLP-1 agonists	SGLT-2 inhibitors	AGIs
		1st generation	2nd and 3rd generation		Rosiglitazone	Pioglitazone				
Relative glycemic efficacy	+++	+++		+	+++		++	+++	++	++
Effect on weight	Loss/neutral	Gain	Gain	Gain	Gain	Gain	Neutral	Loss	Loss	Neutral
Overall CV safety	Benefit	Caution	Neutral	No data	Caution	Likely benefit	Neutral	Benefit	Benefit	No data
Significant side effects	Lactic acidosis	Profound hypoglycemia, secondary failure		Hypoglycemia	Association with increased fracture risk, bladder cancer^a^		GI effects, acute pancreatitis^a^	GI effects	Ketoacidosis, UTI	Flatulence, diarrhea

*TZDs* thiazolidinediones, *DPP*-*4* dipeptidyl peptidase-4, *GLP*-*1* glucagon-like peptide-1, *SGLT*-*2* sodium glucose cotransporter 2, *AGIs* alpha-glucosidase inhibitors, *CV* cardiovascular, *GI* gastrointestinal, *UTI* urinary tract infection

^a^Causative relationship not established

**Table 2:** Suggested preference of pharmacotherapy in type 2 diabetes based on cardiovascular effects

Acceptable	Avoid	No data
*Established or at risk of heart failure*		
Metformin	TZDs	Meglitinides
Sulfonylureas	DPP-4 inhibitors^a^	AGIs
SGLT-2 inhibitors		
*Established cardiovascular disease*		
Metformin	1st generation sulfonylureas	Meglitinides
2nd and 3rd generation sulfonylureas	Rosiglitazone	AGIs
Pioglitazone		
DPP-4 inhibitors		
SGLT-2 inhibitors		

*TZDs* thiazolidinediones, *DPP*-*4* dipeptidyl peptidase-4, *GLP*-*1* glucagon-like peptide-1, *SGLT*-*2* sodium glucose cotransporter 2, *AGIs* alpha-glucosidase inhibitors

^a^Specifically saxagliptin and alogliptin; note that sitagliptin may be used

**Table 3:** Upcoming cardiovascular outcomes trials in type 2 diabetes

Study	Drug	Comparator	Primary endpoint	Results
CANVAS	Canagliflozin	Placebo	Major adverse cardiovascular events, including CV death, non-fatal MI, and non-fatal stroke	2017
CARMELINA	Linagliptin	Placebo	Composite endpoint: cardiovascular death, non-fatal myocardial infarction, non-fatal stroke and hospitalization for unstable angina pectoris	2018
EXSCEL	Exenatide once weekly	Placebo	Composite endpoint: cardiovascular death, non-fatal MI, or non-fatal stroke	2018
REWIND	Dulaglutide	Placebo	Composite endpoint: cardiovascular death, non-fatal MI, or non-fatal stroke	2018
DECLARE-TIMI 58	Dapagliflozin	Placebo	Composite endpoint: CV death, MI or ischemic stroke	2019
CAROLINA	Linagliptin	Glimepiride	Composite endpoint: CV death, non-fatal MI (excluding silent MI), non-fatal stroke and hospitalisation for unstable angina pectoris	2019

*CV* cardiovascular, *MI* myocardial infarction

## Abbreviations

ACCORD: action to control cardiovascular risk in diabetes
AGI: alpha-glucosidase inhibitor
CNODES: Canadian Network for Observational Drug Effect Studies
DPP-4: dipeptidyl peptidase-4
DREAM: diabetes reduction assessment with Ramipril and rosiglitazone medication
ELIXA: evaluation of lixisenatide in acute coronary syndrome
EMPA-REG OUTCOME: empagliflozin cardiovascular outcome event trial in type 2 diabetes mellitus patients
EXAMINE: examination of cardiovascular outcomes with alogliptin versus standard of care
FDA: Food and Drug Administration
GIP: glucose-dependent insulinotropic peptide
GLP-1: glucagon-like peptide-1
HbA1c: mean glycated hemoglobin
LEADER: liraglutide effect and action in diabetes: evaluation of cardiovascular outcome results
MACE: major adverse cardiovascular events
MI: myocardial infarction
NAVIGATOR: nateglinide and valsartan in impaired glucose tolerance outcomes research
K_ATP_: adenosine triphosphate-sensitive potassium channels
PPARɣ: peroxisome-proliferator–activated receptor ɣ
PRESTO: prevention of restenosis with tranilast and its outcomes
PROACTIVE: prospective pioglitazone clinical trial in macrovascular events
RECORD: rosiglitazone evaluated for cardiac outcomes and regulation of glycemia in diabetes
SAVOR-TIMI: saxagliptin assessment of vascular outcomes recorded in patients with diabetes mellitus—thrombolysis in myocardial infarction
SGLT-2: sodium glucose cotransporter-2
STOP-NIDDM: study to prevent non-insulin-dependent diabetes mellitus
SUSTAIN-6: trial to evaluate cardiovascular and other long-term outcomes with semaglutide in subjects with type 2 diabetes
T2DM: type 2 diabetes mellitus
TECOS: trial to evaluate cardiovascular outcomes after treatment with sitagliptin
TZD: thiazolidinedione
UGDS: University Group Diabetes Study
UKPDS: United Kingdom Prospective Diabetes Study
